# A current view of G protein-coupled receptor - mediated signaling in pulmonary hypertension: finding opportunities for therapeutic intervention

**DOI:** 10.20517/2574-1209.2018.44

**Published:** 2018-08-30

**Authors:** Derek Strassheim, Vijaya Karoor, Kurt Stenmark, Alexander Verin, Evgenia Gerasimovskaya

**Affiliations:** 1 Departments of Medicine, University of Colorado Denver, Aurora, CO 80045, USA.; 2 Cardiovascular and Pulmonary Research laboratories, University of Colorado Denver, Aurora, CO 80045, USA.; 3 Department of Pediatrics, Pulmonary and Critical Care Medicine, University of Colorado Denver, Aurora, CO 80045, USA.; 4 Vascular Biology Center, Augusta University, Augusta, GA 30912, USA.

**Keywords:** Pulmonary hypertension, vascular remodeling, vasoconstriction, vascular inflammation, GPCR, intracellular signaling

## Abstract

Pathological vascular remodeling is observed in various cardiovascular diseases including pulmonary hypertension (PH), a disease of unknown etiology that has been characterized by pulmonary artery vasoconstriction, right ventricular hypertrophy, vascular inflammation, and abnormal angiogenesis in pulmonary circulation. G protein-coupled receptors (GPCRs) are the largest family in the genome and widely expressed in cardiovascular system. They regulate all aspects of PH pathophysiology and represent therapeutic targets. We overview GPCRs function in vasoconstriction, vasodilation, vascular inflammation-driven remodeling and describe signaling cross talk between GPCR, inflammatory cytokines, and growth factors. Overall, the goal of this review is to emphasize the importance of GPCRs as critical signal transducers and targets for drug development in PH.

## INTRODUCTION

Pulmonary hypertension (PH) is a complex disease of unknown etiology. The pulmonary circulation responds to hypoxia by vasoconstriction, thereby diverting blood to oxygen rich regions. However, prolonged hypoxic vasoconstriction leads to remodeling of pulmonary arteries (PAs) and increased PA pressure. Increased pressure initially results in compensatory cardiac hypertrophy, but eventually causes de-compensatory cardiac remodeling and death by heart failure. Recent research indicates that PH in all its forms, especially associated with left heart disease, is more common than previously thought^[1]^. Current PH therapies that include endothelin-1 (ET1) receptor antagonists, prostacyclin analogs, cGMP- phosphodiesterase (PDE) inhibitors, and Ca^2+^ channel blockers impede, but do not stop the disease process, emphasizing the need for a finding of alternate treatments^[2]^. Over the years, preclinical research in PH has identified many protein targets, but very few have translated to the bench side. By contrast, G-protein-coupled receptors (GPCRs), the largest superfamily in the genome, play an important role in the development of PH and can be easily targeted by drugs^[3]^. The heart, a prime target for development of new PH therapies, expresses 200 GPCRs^[4]^. GPCR signaling cascades are critical for cardiovascular function and are targeted for the treatment of hypertension and heart failure by agonist and antagonist strategies. Here we reviewed the current knowledge on GPCR signaling in cardiac, vascular, and blood cells and highlighted some critical outcomes in PH, such as vasoconstriction/vasodilation responses, vascular inflammation, vascular and cardiac remodeling, and endothelial dysfunction (ED).

## GPCR-MEDIATED SIGNALING

GPCRs are a family of 7-transmembrane domain proteins, forming a deep binding pocket for the extracellular ligand, agonist, which activates the receptor. Intracellular loops make contact to heterotrimeric G-proteins of 4 different classes (Gα_s_, Gα_i_, Gα_q_, Gα_12_) [Table 1]^[5]^. Agonist binding to GPCRs stimulates GDP/GTP exchange on Gα subunits, converting them into the active state and promote dissociation of G_βγ_ subunits. G proteins interact with multiple effectors, leading to generation of second messengers, including cAMP, 1,2-diacylglycerol, phosphatidylinositol-3, 4, 5-trisphosphate (PIP3), and Ca^2+^. These signaling events are translated into complex hierarchy of kinase network [PKA, PKC, Akt, Ca^2+^/calmodulin-dependent protein kinase (CAMK)] leading to the regulation of gene expression and cellular functions. There are four families of Gα subunits with multiple members. αs exists as multiple transcripts 42 short and 44kD long forms. α_I_ subfamily has α_i1_, α_i2_, α_i3_, α_z_, α_O1_, α_O2_; the α_q_ subfamily has α_11_, α_14_, and α_16;_ and the α_12/13_ family. The β subunits are β_1–5_; and the γ subunits are γ_1–5,7,8,10,11,13_. The βγ subunits, like Gα subunits, activate intracellular effector pathways including MAPK cascades, Rac1, phospholipase C-β (PLC-β, phosphoinositide 3 kinase γ (PI3K- γ, and ion channels and show variation as to the GPCR-Gα -complexes they interact with. Termination of G protein activation cycle occurs by the transition of Gα subunits to GDP-bound state, that is catalyzed by GTPase activating proteins (GAPs), known as regulators of G-protein signaling (RGS proteins). There are 31 proteins, containing the RGS domain that function as GTPase enzymes, terminating G-protein signaling^[6,7]^.

## GPCR SIGNALING IN VASOCONSTRICTION AND VASCULAR REMODELING

**Figure 1.**
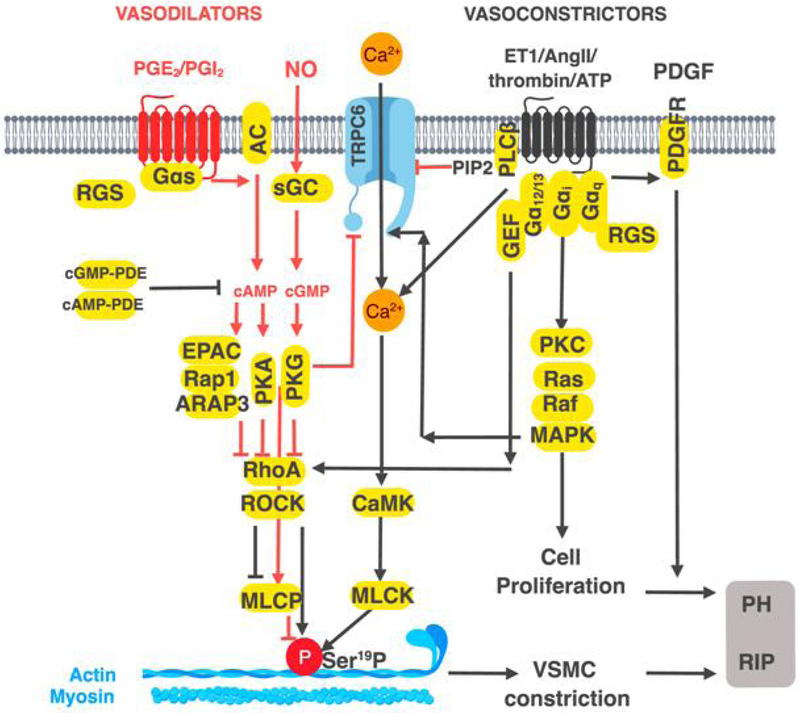
Schematic presentation of the mechanisms by which G protein-coupled receptors (GPCRs) regulate vascular tone and vascular smooth muscle cells (VSMC) proliferation. Vasoconstrictors like Ang II, ET1, thrombin, activate Gα_i_, Gα_q_, or G_12_/_13_-coupled GPCRs, increase Ca^2+^ via PLC_β_ activity, and receptor operated calcium channels such as TRPC6. Increase in PLC_β_ activity decreases PIP2 relieving tonic inhibition of TRPC6. Increase in Erk1/2 activity by G_i_/G_q_-coupled GPCRs activates TRPC6 by phosphorylation leading to increased Ca^2+^ entry and calmodulin-dependent protein kinase (CAMK) activation. CAMK increases MLCK activity by phosphorylation, which in turn phosphorylates MLC phosphorylation causing vasoconstriction. GPCRs coupled to G_12_/_13_ increase RhoA activity and the downstream kinase ROCK. ROCK increases MLC phosphorylation by inhibiting MLCP, or by direct phosphorylation. Vasodilators, such as PGI_2_ acting via G_s_-coupled receptors activate PKA thereby inhibit Ca^2+^ increase by PKA-mediated phosphorylation of PLC_β_ and TRPC6. In ECs, G_i_, or G_q_-coupled GPCRs, increase, PI3K-Akt signaling and activate eNOS by phosphorylation at Ser^1177^. NO diffuses to nearby VSMC, activating soluble guanylate cyclase, increasing cGMP, activating PKG, and inhibiting TRPC6 by phosphorylation. PKG also activates the GAPs for G_q_, RGS2 and RGS4 to inhibit PLC_β_ activity thereby attenuating Ca^2+^ entry. Both PKG- and PKA inhibit RhoA by direct phosphorylation and promote vasodilation

Vasoconstriction is driven by Ca^2+^-dependent phosphorylation of myosin light chain (MLC) on Ser^19^-MLC, whereas vasodilators oppose this event^[8–10]^ [Figure 1, Tables 1 and 2]. In vascular smooth muscle cells (VSMC), the vasoconstrictor response is mediated by G_i-_, G_q-_, or G_12/13_-coupled GPCRs for ET1, angiotensin II (Ang II), serotonin, and thrombin^[11–16]^. G_i_ and G_q_ activate PLC pathways, increasing Ca^2+^ and receptor operated calcium entry (ROCE) via transient receptor potential cation channel subfamily C member 6 (TRPC_6_) channels. TRPC_6_-activation occurs by several mechanisms, including direct ERK1/2-mediated phosphorylation of TRPC6. Secondly, phosphoinositide-4, 5-bisphosphate (PIP_2_), the substrate for PLC, is an inhibitor of TRPC_6_^[17,18]^. Activation of G_12/13_ by vasoconstrictor GPCRs stimulates G_12/13_-dependent RhoA GEFs to increase the activity of, RhoA. In turn, RhoA activates Rho associated kinase (ROCK), which leads to increased Ser^19^-MLC and thereby, vasoconstriction^[19,20]^. Vasodilators, such as prostaglandin I_2_ (PGI_2_), acting via G_s_-coupled (IP) receptor on VSMC, activate PKA and decrease intracellular Ca^2+^, leading to reduced MLC phosphorylation on Ser^19^ [Figure 1, Table 1].

Vasodilators decrease intracellular Ca^2+^ by inhibiting PLCβ and TRPC_6_. The mechanism involves PKA/PKG-mediated phosphorylation of PLCβ and TRPC_6_ (on Ser^28^) and by phosphorylation of RGS4, which inhibits G_q_-dependent activation of PLCβ^[21–23]^. Vasodilator GPCRs that increase cAMP may also activate cAMP-binding domain in exchange factor EPAC1, a GEF for the small molecular weight G-protein Rap1, a member of Ras superfamily. Rap1 activates ARAP3, a Rho GAP, which in turn, inhibits RhoA, leading to reduced MLC phosphorylation and vasodilation^[24,25]^. Vasodilation also occurs via endothelial cell (EC)-dependent production of nitric oxide (NO) by endothelial nitric oxide synthase (eNOS), which is activated by Akt or ERK1/2 by phosphorylation on Ser^1177^ residue^[26]^. Highly permeable NO readily enters VSMC, stimulates soluble guanylate cyclase (sGC) and activates cGMP-PKG, antagonizing Ca^2+^ action on phosphoSer^19^-MLC and promoting vasodilation. More specifically, NO-sGC-cGMP-PKG-axis inhibits Ca^2+^ increase by stimulating TRPC6 phosphorylation at Thr^69^, decreasing ROCE and increasing vasodilation^[27]^. PKG phosphorylates and activates RGS2, and RGS4, that leads to the inhibition of G_i_/G_q_,-rergulated PLC activity and termination of the vasoconstrictor Ca^2+^ signal^[23]^. Both PKG and PKA phosphorylate and inhibit RhoA and increase the activity of myosin light chain phosphatase (MLCP), thereby decreasing MLC contraction^[28,29]^. MLCP is also activated by vasodilators by PKG-mediated phosphorylation of a MLCP inhibitory subunit^[20]^. In addition, PKG and PKA reduce the ability of RhoA to inhibit the delayed rectifier potassium channel (KDR), which attenuates extracellular Ca^2+^ entry^[30]^. The enzyme PDE5A, a target of sildenafil therapy in PH, hydrolyzes cGMP to counter the effects of NO-cGMP-PKG signaling. However, other PDEs, including cAMP PDEs, play important roles^[31]^. Vasoconstrictors activate PDE5A to reduce cGMP in VSMC by RhoA/PKC-mediated inhibition of protein phosphatase 1 (PP1), thereby increasing phosphorylation of PDE5A and activating it^[32]^. GPCRs, including those for adenosine, ATP, adiponectin, apelin, prostaglandin E2 (PGE2,), PGI_2_ generally increase NO from EC, which diffuses to VSMC, or directly increase cAMP in VSMCs^[33–39]^.

As a final summation statement, all current PH therapies intersect GPCR actions by modulating critical signaling effects. Firstly they, ultimately inhibit intracellular Ca^2+^ signaling and vasoconstriction. This includes the cGMP-PDE inhibitors, soluble guanylate cyclase (sGC) activators, PGI_2_ analogs, Ca^2+^-channel blockers, and ET-1 receptor antagonists. Secondly, they exert anti-inflammatory effects on vascular cells, as all of these therapeutics are known to do^[2,40,41]^.

### GPCR ligand-dependent vasoconstrictor response

Vasoconstrictor ligands, including ET-1, TxA_2_, and serotonin are increased in serum of PH patients; for serotonin a 4–5 fold increase has been reported, (8.8 ± 0.6 nmol/L) *vs.* (38.8 ± 7.3 nmol/L)^[42–47]^. Serotonin, acting via 5-HT_1B_-G_i_ coupled and 5-HT_2A/2B_-G_q_ coupled GPCRs, stimulates VSMC proliferation via the activation of the transcription factor GATA-4 and increase of cytokine generation from leukocytes, such as dendritic cells^[48]^. TxA_2_ level in PH is elevated due to up-regulation of thromboxane-A synthase^[46]^. Increased presence of inflammatory cytokines, such as TNFα and IFNγ, stimulates ET1 release from VSMC, believed to be an important source of the vasoconstrictor ET-1 in PH. This effect of cytokines and ET1 is antagonized by the PGI_2_-IP axis^[49]^.

### GPCR ligand-dependent vasodilator response

In contrast to vasoconstrictors, several vasodilators are decreased in PH, promoting vasoconstriction in pulmonary vascular system. Apelin, the ligand for CVD protective GPCR (APJ), modestly falls in PH patients (1.25 ng/mL *vs.* 0.89 ng/mL, *P* = 0.037)^[50–52]^. Decreased PGI_2_ synthase (PGIS) in ECs also plays a role in vasodilation and inflammation^[45,46]^.

### Increased activity of vasoconstrictor GPCRs

GPCR activity is frequently altered in diseases via internalization, phosphorylation, and expression levels. In lung, increased activity of TxA_2_ and its G_q_-coupled GPCR (TP) occurs via palmitoylation of TP and increasing the proportion of the active receptor at the plasma membrane, consistent with pathophysiological action of TP in PH^[53–56]^. Similarly, increased expression of other GPCRs involved in PH pathogenesis has been noted for ET1 (ET_A_) and serotonin receptors, 5-HT_1B_R and 5-HT_2B_R in COPD-PH patients^[54,55,57,58]^.

### Decreased activity of vasodilator GPCRs

In PH, decreased serum concentrations of PGI_2_ is accompanied by decrease in levels of the receptor IP, reducing the effectiveness of PGI_2_ therapy^[59]^. Similarly, chronic stimulation of PGI_2_-IP axis, occurring with prostacyclin therapy in PH patients, is likely to even further down regulate the PGI_2_-IP axis via heterologous desensitization, compounding a pathogenic situation^[60–62]^. GPCRs such as IP, which increase cAMP-PKA, frequently exert anti-inflammatory effects, inhibiting key pro-inflammatory/pro-proliferative transcription factors, including NF-κB^[63,64]^, Hippo pathway transcription factors Yaz-Taz (co-factors for the pro-proliferative transcription factor TEAD1) and, no doubt, many others^[65]^. Induction of anti-inflammatory/anti-proliferative PPAR_γ_ is also another mechanism, by which PGI_2_ acts^[66]^. PPAR_γ,_ along with sibling, transcription factors PPAR_β_/_δ_ all are protective in PH and other cardiovascular diseases^[34,66–71]^. The induction of PPARγ activity by PGI_2_ was once thought to be a direct binding event to the PPARγ, but it now appears to occur by indirect mechanism. Activation of PKA or p38MAPK by PGI_2_-IP stimulates the cAMP response element-binding protein (CREB) by phosphorylation. Activated CREB turns on the transcriptional co-activator, peroxisome proliferator-activated receptor gamma co-activator 1α (*PGC1*α) gene, increases PGC1a activity and stimulates PPARγ, leading to protective anti-inflammatory effects^[71]^ Molecular targets of PPARγ include inhibition of NF-κB and hypoxic activation of HIF-1α^[72]^. HIF-1α is clearly important in VSMC proliferation occurring in PH, as it helps the cell switch to a glycolytic/Warburg metabolic phenotype and has been connected to the increased expression of Ca^2+^ entry channel, TRPC6, both aiding VSMC proliferation^[73–76]^. Targeted KO of HIF-1α inhibitor protein, prolyl-hydroxylase domain containing protein 2 (PHD2), reduced O_2_-driven proteolysis of HIF-1α, thereby increasing HIF-1α -dependent proliferation of VSMC^[76]^. There are 3 PHD (PHD1–3) enzymes, which in presence of O_2_ hydroxylate proline residues, 402 and 564, ultimately resulting in the proteolysis of HIF-1α. A small molecule drug, R59949, a PDH inhibitor, has shown potential to combat PH in the hypoxic mouse model^[76]^.

### Post-receptor mechanisms leading to increased vasoconstrictor GPCR response

In VSMC, Angiotensin II (Ang II) up regulates G_i_ expression, thereby increasing the activation of PLCβ and mobilization of Ca^2+^, further enhancing vasoconstriction and proliferation by a post-receptor mechanism^[77]^. Of the PH pre-clinical therapeutics, RhoA-ROCK inhibitor, fasudil and statins both act at post GPCR level^[78,79]^. Statins, such as simvastatin, can work in combination with sildenafil, the cGMP-PDE inhibitor, likely an important feature of any new therapy. Although some studies reported no drug combination yet tested, the combination could be more effective for patients’ survival than any monotherapy^[2,80,81]^. Statins may work in PH models by inhibition of isoprenoid intermediates, farnesyl pyrophosphate and geranyl-geranyl pyrophosphate, essential for the post-translational isoprenylation, membrane localization, and activation of Ras and Rho small GTP-binding protein families, respectively, thus inhibiting RhoA-ROCK^[82]^.

### Post-receptor mechanisms leading to decreased vasodilator GPCR responses

Post-receptor mechanisms also operate to limit vasodilator response in PH, such as the several hits to the critical NO-cGMP-PKG vasodilation system. Firstly, inflammatory cytokines down regulate eNOS and upregulate reactive oxygen species (ROS), including superoxide^[83–85]^. Secondly, due to peroxynitrite formation, NO level is depleted^[86]^. Thirdly, vasodilator response can be limited due to increased PDE5_A_ expression^[87,88]^. Up regulation of both cAMP-PDEs, and cGMP-PDE is an important pathological event, which decreases effectiveness of vasodilator GPCRs and needs further investigation^[89]^. The PDEs are a complex family of enzymes with 21 genes, and 11 subfamilies, and some share little sequence identity^[31]^. Due to a combination of post-receptor mechanisms, increased expression of cAMP- and cGMP-PDEs, inhibition of eNOS activity, and decreased NO availability (as a result of ROS production), the effects of vasodilators in PH are diminished.

## HOW GPCRS FUNCTION IN VASCULAR INFLAMMATION-DRIVEN REMODELING

GPCRs induce cytokine/chemokine production from leukocytes, VSMC, ECs, fibroblasts, and cardiac myocytes and are pathogenic in PH. Up regulation of SDF-1 in activated T cell results to the expression and secretion of RANTES and Monocyte Chemo-attractant protein 1 (MCP-1). These chemokines promote proliferation of VSMC, matrix remodeling, and ROS production^[90–92]^. Additionally, GPCRs like serotonin receptor and purinergic P_2_Y_14_R, promote migration of bone marrow derived blood cells, essential to the development of PH^[93,94]^.

## DAMAGE MOLECULAR PATTERNS AS A POTENTIAL CONTRIBUTOR TO VASCULAR INFLAMMATION IN PH

**Figure 2.**
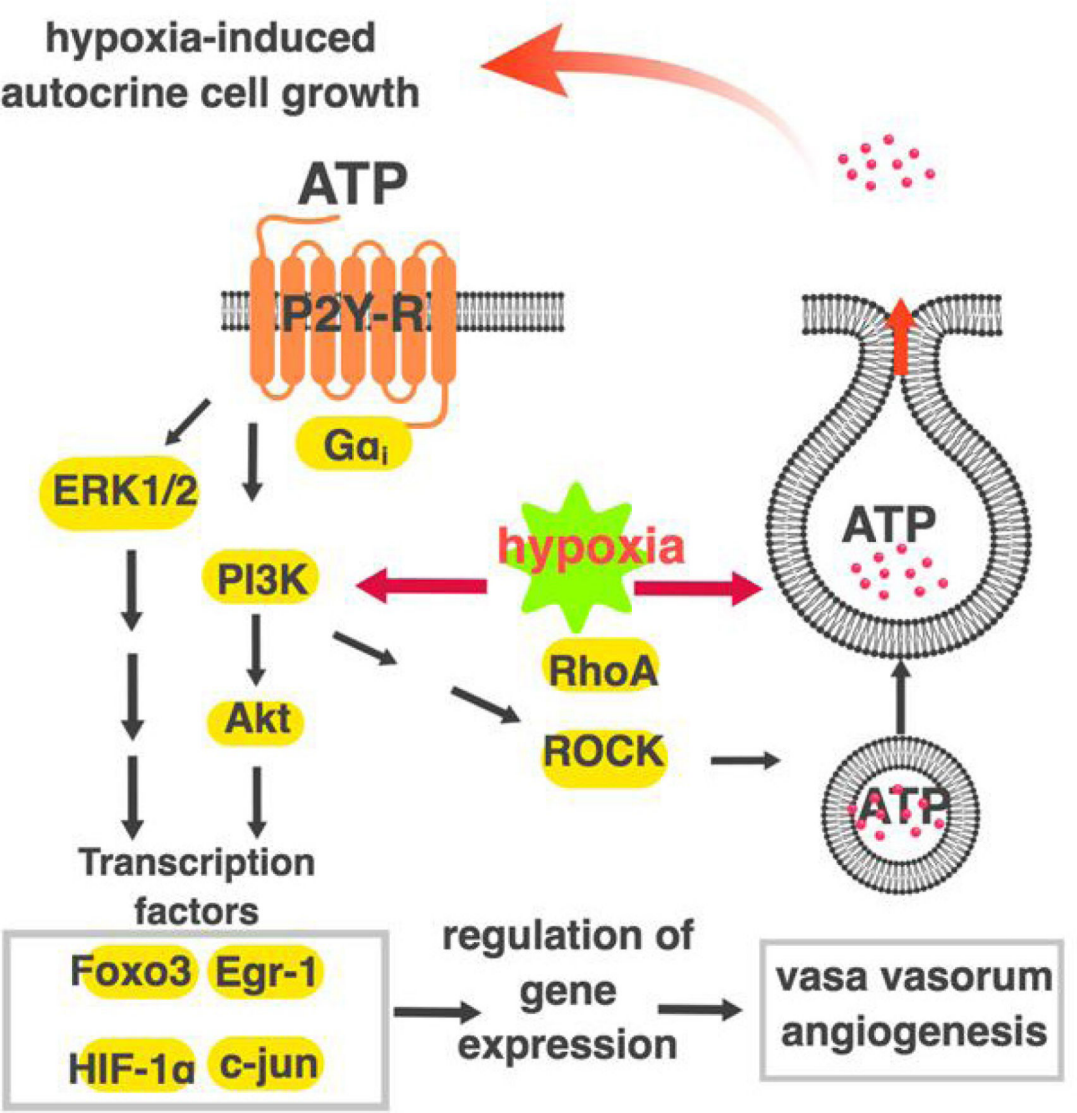
Schematic diagram illustrating a role of PI3K, Rho and ROCK pathways in hypoxia-induced ATP release and ATP-mediated angiogenic effects in vasa vasorum endothelial cells. Activation of PI3K/Rho/ROCK pathway in response to hypoxia results in regulated ATP release from VVEC. In turn, extracellular ATP triggers/initiates P2YR-dependent activation of PI3K/Rho/ROCK pathway leading to angiogenic responses in vasa vasorum endothelial cells. VVEC: vasa vasorum endothelial cells

The driving forces behind vascular inflammation in PH are unclear, but it is likely that sterile inflammation-damage molecular pattern (DAMP) systems play a role. Purinergic receptors are also critical in DAMP responses. ATP, ADP, or adenosine are released from extracellular stimuli-activated, hypoxic, or damaged cells and play prominent roles in inflammatory and secretory responses associated with tissue repair. Of the 19 purinergic receptors, 12 are GPCRs nucleotide P2YR_1, 2, 4, 6, 11–14_ and adenosine A_1_, A_2A_, A_2B_ A_3_, and the remaining 7 purinergic receptors P2X_1–7_, are ligand gated cation channels^[95–100]^. Macrophage activation in PH is potentiated by the P_2_Y_6_^[101–103]^. Some data suggest antagonizing the ATP-activated P_2_X_1_ purinergic receptor could be beneficial in PH^[104]^. Both P_2_Y_1_ and P_2_Y_12_ purinergic receptors have been shown to be partially responsible for PA pressure increase due to hypoxia^[105]^. Hypoxia-induced ATP release from PA adventitial fibroblasts and vasa vasorum endothelial cells (VVEC) induces mitogenic and angiogenic responses in VVEC in autocrine/paracrine manner^[95,96,106]^ [Figure 2]. Released ATP and ADP are degraded rapidly to adenosine. Activation of the A_2A_ adenosine receptor has been reported to be protective against PH, but the activation of A_2B_-AR results in pathogenic effects^[107–112]^. The involvement of DAMPS-GPCRs in PH is understudied, and therapeutic possibilities remain to be explored.

## PATHOGENIC CHEMOKINE GPCRS

### Small G-proteins in chemokine receptor-stimulated VSMC proliferation

In VSMC, MCP-1 acting via G_i_-coupled CCR2, stimulates G_i_-dependent proliferation, that also involves activation of the small G proteins^[113]^. One of the mechanisms includes p115RhoGEF-dependent activation of the Rac and Nuclear factor of activated T-cells (NFAT1)-dependent up-regulation of cyclin D1 expression in VSMC^[113]^.

### Involvement of ROS in chemokine receptor-stimulated responses

ROS is a pathogenic factor in PH by mechanisms, which include reducing NO; promoting VSMC proliferation; initiating sterile inflammation-DAMP response; and promoting vasoconstriction via increased membrane depolarization^[74,114]^. G_i_-coupled GPCRs, such as MCP-1, SDF-1, thrombin, PAF, and purinergic receptors, stimulate ROS production^[115–117]^. ROS are produced as bactericidal compounds in large amounts in phagocytes (neutrophils, monocytes, macrophages) and, in a lesser amounts, in vascular cells. In phagocytes, chemokines, such as N-Formylmethionyl-leucyl-phenylalanine, PAF, complement C5a (C5a), LTB_4_, and MCP-1 are G_i_-coupled-GPCRs and activate Rac1-NAD(P)H oxidase-superoxide system. NOX2 is a neutrophil NADPH oxidase responsible for producing increased amounts of superoxide. There are 7 NOX like oxidases, NOX1–5 DUOX1, 2 of which are expressed in vascular cells, and their activation involves Rac1 stimulation by the GEFs, such as engulfment and cell motility protein 1 (ELMO1)^[115,117,118]^. The superoxide generated by NOX enzymes in the extracellular space, is converted to H_2_O_2_, some of which enters the cell to stimulate proliferation. H_2_O_2_ induces proliferation by changing the balance in protein kinase-protein phosphatase networks by inhibiting key protein phosphatases via the oxidation of labile sensitive cysteine in the active site^[119]^.

### The involvement of HIF-1α in chemokine/GPCR action with respect to PH

**Figure 3.**
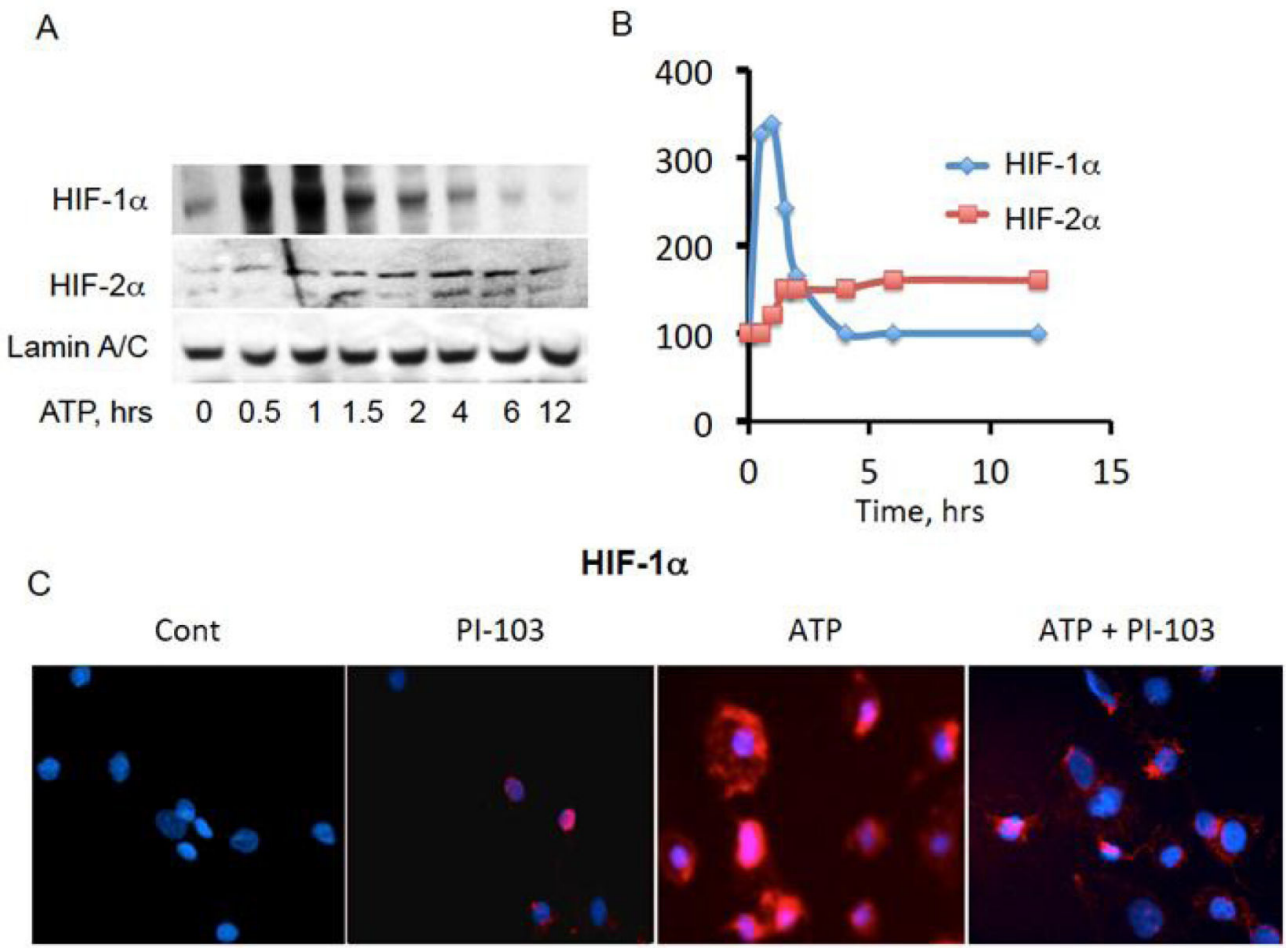
Extracellular ATP up regulates HIF-1_α_ and HIF-2_α_ transcription factors in pulmonary artery vasa vasorum endothelial cells. A, B: ATP (10 _μ_mol/L), applied to VVEC, results in activation of both HIF-1_α_ and HIF-2_α_ with distinct time courses. VVEC were serum starved for 18 h and stimulated for indicated times. Nuclear fractions were subjected for Western blot analysis for HIF-1_α_, HIF-2_α_, and lamin A/C expression; C: cells were stained for HIF-1_α_ at 1 h post stimulation with ATP (10 _μ_mol/L), with or without PI3K inhibitor, PI-103 pretreatment (0.5 _μ_mol/L, 15 min). VVEC: vasa vasorum endothelial cells

HIF-1α and HIF-2α may play a pathophysiological role in PH, and the action of GPCRs overlaps with that of HIFs^[76,120,121]^. Firstly, some GPCRs, such as those for estrogen G-protein coupled estrogen recetor-1 (GPER), ET1 (ET_A_), PGE_1_ (EP_1_), and PGI_2_ (IP), can activate HIF-1α even under normoxic conditions^[122–131]^. Secondly, ROS increased by GPCRs signaling, inhibit PHD proteins by oxidative inactivation, which in turn promotes HIF1α activation and its pathological action in PH^[132–135]^. Thirdly, hypoxic activation of HIF1α up regulates G_i_-coupled receptor for SDF-1, CXCR4, implicated in PH by promoting VSMC proliferation^[136–139]^. Moreover, hypoxia can stimulate ATP release from vasa vasorum endothelial cells (VVEC) by PI3K-dependent mechanism to promote angiogenesis in an autocrine manner [Figure 2]. This mechanism implicates purinergic GPCR-dependent activation of HIF-1α and HIF-2α that may amplify hypoxia-induced vasa vasorum expansion [Figure 3].

## INTERACTION OF INFLAMMATORY CYTOKINES AND GROWTH FACTORS WITH GPCRS SIGNALING IN PH

PDGF-induced proliferation of VSMC is believed to be a major factor in PH. It is known to be dependent on Akt activation that can occur in co-operation with some GPCRs, termed trans-activation^[140]^. Ang II receptor works in concert with PDGF-receptor tyrosine kinase, promoting Akt-dependent VSMC proliferation^[77,141–143]^. Thrombin-PAR trans-activates the TGF-β receptor to promote VSMC proteoglycan synthesis^[144]^. It is of some interest that PGI_2_ has been described as unable to significantly inhibit PDGF-induced VSMC proliferation, suggesting that other PDGF-neutralizing strategies are needed in PH^[145]^. MCP-1 and IL-6 also work together to induce VSMC proliferation^[146]^. Activation of inflammatory TXA_2_-TP inhibits FGF-2- or VEGF-stimulated angiogenesis, which could relate to vascular pruning in cardiac and pulmonary vessels, and is an example of GPCR-cytokine interaction^[41,147–149]^. Protective interactions of GPCRs with cytokines and growth factors could include the ability of PGI_2_-IP to inhibit the IFNγ-induced inflammation, dependent upon induction of suppressor of cytokine signaling 3 (SOCS3)^[150]^. The GPCR GPR4 expressed on ECs, promotes angiogenesis in a Notch-dependent manner^[151]^. Vessel architecture is maintained by the ligand-receptor pair jagged expression on EC and Notch expression on VSMC, keeping VSMC in a differentiated non-proliferating state^[152–156]^. Both HIF-1_a_-induced VEGF for reparative angiogenesis and hypoxia-induced epithelial to mesenchymal transition require Ras family member, RhoE, which activation involves SDF-1 GPCR, CXCR4 signaling^[157]^. RhoE aids in HIF-1α maintenance and is induced by cAMP via G_s_-coupled GPCRs^[158]^. Cardiac angiogenesis is believed to be critically protective in heart disease and potentially links SDF-1, cAMP, RhoE, HIF-1α, and VEGF into signaling networks^[159]^.

## INTERSECTIONS OF EICOSANOIDS AND GPCRS IN VASCULAR INFLAMMATION

Many eicosanoids induced by vascular inflammation, have short half-lives and must therefore be produced at the site of action either by monocyte/macrophages, ECs, fibroblasts, cardiac myocytes, or fibroblasts^[160,161]^. Injection of the GPCR-G_q_/G_i_-coupled ligand, PAF into rat lung causes rapid increase in PA pressure, linked to LTB_4_ production. LTB4-LTB4R, and PAF-PAFR coupled G_q_/G_i_ are macrophage activators and plays a pathological role in PH^[162–169]^. PGE_2_, an important eicosanoid, which activates several GPCRs, such as G_q_-coupled EP1, G_s_-coupled EP_2/4_ and Gα_i_/Gα_13_-coupled EP_3_. EP_3_ promotes PH by increasing Rho/TGF-β1 signaling^[170]^. Protective eicosanoids, like PGI_2_, exert anti-inflammatory effects following LPS-induced lung injury and PH-induced cardiac inflammation and is active against T cells and macro-phages[41,171–174].

## INFLAMMATION-DRIVEN ENDOTHELIAL DYSFUNCTION (ED) AS A MECHANISM OF VASCULAR REMODELING: INVOLVEMENT OF GPCRS

Inflammatory stimuli, IL-1 or TNFα down-regulate eNOS, attenuate reparative angiogenesis, promote EC apoptosis, and increase endothelial to mesenchymal transition (EMT) - all of which contribute to ED^[46,83,175,176]^. TxA_2_, acting on both ECs and VSMCs, is pathological in PH and inhibits VEGF- or FGF-2-promoted angiogenesis^[46,147–149,165]^. By contrast, many PH protective GPCR agonists (apelin, PGI_2_) increase eNOS activity by phosphorylation of Ser^1177^ or by increasing eNOS expression^[50–52,177–179]^. Some PH therapeutics, apelin and sildenafil, increase recruitment of endothelial cell progenitors, thereby counteracting ED^[180–184]^.

## THROMBOSIS AND PLATELET ACTIVITY CROSS TALK WITH VASCULAR INFLAMMATION AND GPCR ACTION

Platelets from patients with the sub-form of PAH, due to thromboembolic PAH, exhibit increased reactivity to thrombin, which stimulates the G_q_/G_i_-coupled protease activated receptor 1 (PAR1), promoting VSMC proliferation^[185,186]^. Thrombin receptors exist on EC and have been reported to inhibit angiogenesis.

## RV REMODELING AND FAILURE

Cardiac myocytes (CMs) are terminally differentiated cells. The compensatory cardiac hypertrophy is entirely due to increased CM cell size, rather than proliferation. The adult heart is 56% CM, 27% fibroblasts, 10% VSMC, and 7% ECs, and these ratios change little between the four chambers^[187]^. During PH, the ratios of fibroblasts increases, and the ratio of ECs/CMs decreases^[188]^. The transition to heart failure has been linked to endothelial dysfunction due to insufficient reparative angiogenesis - a loss of capillaries supplying cardiac myocytes with O_2_, leading to capillary pruning, inflammation, and ROS production^[147–149,188–193]^.

### Pathological role of GPCRs in cardiac myocyte with respect to RV failure

The hypertrophy response is engaged when increased Ca^2+^- and cAMP-dependent contractile signals lead to activation of NFAT, MEF2, and GATA_4_. These signals are driven by GPCR agonists, such as Ang II, thrombin, ET1, PGF2α_,_ β-AR^[194–197]^. Typical gene expression changes include decreased expression of sarcoplasmic reticulum Ca^2+^ re-uptake channel (SERCA2), increased expression of slow twitch contractile protein myosin heavy chain β9 (β-MHC, *a.k.a.* MyH7), and decreased expression of the fast twitch a-MHC/MyH6, amongst others^[198,199]^. The transcription factor, Egr-1 has been linked to the down regulation of cardiac SERCA2 in hypertrophy and was found to be overexpressed in PAs of PH patients^[200–202]^. GPCR-induced increase in intracellular Ca^2+^ stimulates PKD activity, promoting nuclear export of histone deacetylase 5 (HDAC5), thereby activating MEF2 to initiate hypertrophic gene program^[203,204]^. GPR91, a receptor for succinate expressed in CMs, promotes cardiac hypertrophy by coupling to G_i_/G_q_-PI3K-Akt signaling^[205,206]^. Succinate may be accumulated during cardiac remodeling due to changes in metabolism, and when released from the cells, promotes positive feedback loop by activating GPR91 leading to hypertrophy, or as also reported, to CM apoptosis via caspase3^[188]^.

### Protective role of GPCRs in cardiac myocyte with respect to RV failure

The estrogen-activated GPER, found in CM, has been considered cardio-protective in a PI3K-Akt-dependent mechanism^[207,208]^. RGS proteins 2, 4, 10, 14 modulate cardiac hypertrophy by inhibiting the G_i_/G_q_PLCβ-Ca^2+^ signaling axis. PKG activates RGS2 by phosphorylation, inhibiting G_s_, G_q_, and G_i_ signaling, which in turn, attenuates β-AR-induced hypertrophy and that of other GPCRs^[209–212]^. Atrial natriuretic peptide (ANP) and brain natriuretic peptide (BNP) exert CV protective actions by the activation of cGMP-dependent PKG, which phosphorylates and activates RGS4, aiding its inhibition of GPCR-G_q_-PLCβ-Ca^2+^ axis^[213]^. RGS6 promotes cardiac myocyte apoptosis associated with decompensation due to its capacity to increase ROS^[214]^. RGS10 inhibits the cardiac hypertrophy induced by Ang II^[215]^. RGS14 protects against aortic banding-induced cardiac hypertrophy and fibrosis, decreasing ERK1/2 hypertrophy signals^[216]^.

## ACTION OF GPCRS ON ENDOTHELIAL CELLS WITH RESPECT TO RV FAILURE

ED, occurring in failing RV, interconnects with fibrosis, as this appears to be a factor in the decreased capillary density-ED observed in hypertrophy and with the altered metabolism of CM, critical towards HF^[217,218]^. ED can result in potentially uncontrolled inflammation of local RV tissue and in turn can lead to EC apoptosis, down regulation of eNOS and PGIS. TGF-β, which is pathologic in PH, is induced by inflammation, promotes lung and heart fibrosis, but also promotes ED by inhibiting differentiation of endothelial progenitor cells (EPCs) into ECs to repopulate damaged endothelium, counteracting the effects of endothelium protective GPCR ligand, apelin^[219,220]^. Cardiovascular protective GPER is found in ECs, promotes angiogenesis, and could be significant in defending against endothelial dysfunction^[207,221,222]^.

## VASCULAR FIBROBLASTS AND CARDIAC FIBROSIS

Cardiac fibrosis, seen in animal models of PH, involves expansion of fibroblast populations, their differentiation to myofibroblast, and the stiffening of the extracellular matrix by synthesis of collagens^[198]^. Fibroblasts also can derive from EMT via conversion of EC to fibroblasts^[175]^. GPCRs promoting cardiac fibrosis include G_q_-PLC-Ca^2+^- coupled 5-HT_2B_, Ang II, and endothelin CPCRs. The thrombin receptor, PAR1 is the most highly expressed GPCR in cardiac fibroblasts, therefore is a potentially important profibrotic GPCR^[223–225]^. P_2_Y_6_-purinergic receptors are reported to enhance pressure overload-induced fibrosis by increasing TGF-β1 and CTGF release^[226]^. The p38_a_ MAPK, activated by Ang-II or non-GPCR stimuli, such as TGF- β1, or cyclic stretch, has been identified as a master switch, common to many different receptors stimulating fibrosis^[198]^. The ligand relaxin and its GPCR, RFXP1–4, are Gs-coupled and exert anti-hypertrophic and anti-fibrotic effects^[227]^. In cardiac fibroblasts, PGI_2_-IP-PKA axis activates CREB to inhibit Ang II-induced SMAD2 activation, attenuating proliferation^[228]^.

## ROLE OF GPCRS IN MONOCYTE/MACROPHAGE WITH RESPECT TO RV FAILURE

Macrophage features in the inflammation associated with heart failure, with resident macrophages being described as protective, while recruited being pathogenic^[191]^. Increasing activity of the transcription factor KLF4 in resident macrophages to aid their survival or inhibiting MCP-1-CCR2 activity of recruited monocytes, has been suggested as a potential therapy^[191]^. Macrophage polarization in PH is thought to contribute to cardiac and pulmonary inflammation-induced damage and remodeling. M1 macrophage phenotype is considered pro-inflammatory (versus the M2 phenotype), is involved in resolving inflammation, but implicated in tissue fibrosis^[229]^. Some studies in PH suggest that M2 macrophages are more damaging than M1. Antagonizing the CX3CR1 chemokine receptor reduces pathogenic M2 in favor of less damaging M1 phenotype^[90,230]^. Most chemokine receptors activate Gα_i1_/Gα_i3_, which have been linked to promotion of polarization to M1 macrophage via increased LPS-TLR4-NF-κB, in contrast to CX3CR1 signaling^[76]^. An interesting development in macrophage polarization/anti-inflammatory responses are the 6 atypical chemokine receptors, ACKR1–6, which are “duds” unable to activate G-proteins, and exert anti-inflammatory effects^[229]^. In particular, the atypical chemokine receptor, CCRL2 (tentatively ACKR5) polarizes in favor of M2 phenotype^[229]^. Other GPCRs aiding polarizing to M2 phenotype, include lipoxinA4-activated FPR2, PGE_2_-receptors, and adenosine A_2A_/A_2B_-receptors^[231–234]^. GPCRs clearly critically control macrophage polarization and might well be employed to diminish macrophage-induced inflammation occurring in PH. The role of GPCRs in cardiac inflammation is clearly complex, and it should be mentioned that increasing recruitment of pro-angiogenic monocytes may be beneficial in ED, and is also under control of GPCRs^[235–238]^.

## GPCRS, WHICH MIGHT BECOME CLINICAL TARGETS IN PH

GPCRs activating cAMP-PKA axis in ECs or VSMCs, such as PGI_2_ and adenosine (A2_B_AR), generally induce vasodilation, are often anti-inflammatory and protective in PH. Secondly, GPCRs, such as for apelin, PGI_2_, opioids, which increase NO release from EC to promote vasodilation, are also usually protective. Thus, any signals increasing cAMP, cGMP, NO and inhibiting Ca^2+^ are usually protective^[178,179]^. By contrast, any GPCR signaling increasing Ca^2+^ in VSMC, or decreasing NO, cAMP, cGMP, or increasing inflammation, are usually pathogenic in PH. One very potent anti-inflammatory agent is adenosine, which exerts powerful anti-inflammatory effects acting at A_2A_AR, and clearly plays a protective role in PH^[111,239]^. New drugs (such as AEA061) are positive allosteric modulators of A_2_AAR, that activate receptors without binding to the normal agonist binding site, offer a therapeutic possibility of fewer side effects as they do not act at A_1_, A_2B_ or A_3_ARs^[239]^. Activation of A_2A_AR without activating A1, A2B, and A3ARs has been an issue in developing anti-inflammatory therapies. Other potentially protective GPCRs include FPR2, an atypical chemokine receptor on macrophages, was reported to exert anti-inflammatory action^[229,240]^. Other protective receptors in PH include ET-1 receptor ET_B_^[241]^, angiotensin II type 2 receptor^[242]^, adiponectin-receptor^[36,243]^, mas1 (a receptor for angiotensin 1–7)^[244]^, and relaxin receptors^[245,246]^. ET_B_ receptor is also protective in porto-pulmonary hypertension, a disease secondary to liver failure, but in which the same therapeutics, PGI_2_-cGMP-PDE-ET-1 receptor antagonist therapies are utilized^[247,248]^.

GPCRs with pathogenic action, which could be antagonized such that the drugs would be protective could include the CaSR, calcium sensing receptor in EC^[12,249]^, the succinate GPR91 on cardiac myocytes^[205,206]^, thromboxane receptors^[250]^, serotonin receptors^[251]^, LTB_4_ receptors^[252]^, shingosine-1-phosphate receptors^[13,253–255]^ amongst others.

## CONCLUSION

Research has highlighted many examples of pathological GPCR signaling, which can be targets for novel PH therapeutics. In PH pre-clinical studies many targets have been identified, but only few are druggable [Tables 1 and 2]. GPCRs, by contrast, represent good targets for pharmacological strategies and in all likelihood present one of the best opportunities for therapeutic intervention in PH. The heart alone is estimated to express some 200 different GPCRs, suggesting significantly better therapeutics based on targeting GPCRs are possible. The challenge is to devise the best pharmacological cocktail for the PH patient. At the moment, while much has been published with respect to GPCR action in PH, much more clearly awaits discovery.

## Figures and Tables

**Table 1.** G protein-coupled receptor physiology and pathology in pulmonary hypertension

Physiology	Ligand-receptor-reference	Cell	G-protein	Important pathways	PH pathology
Vasodilation	Adenosine-A_2A_-AR; PGI_2_-IP^[110–112]^	VSMC	G_s_	PKA	+
EC-eNOS-NO dependent vasodilation	Adenosine-A_2A_-AR; ApelinAPJ; Relaxin-RXFP; Opioid-KOR^[50,51,66,110–112,178,179,182,245,246]^	EC	G_i_	PKG	+
Vasoconstriction	ET1/ET_A_; Ang II-AT1; TXA_2_-TP; PAF/ PAFR; Shingosine-1-P/S1P_1–5_; Ca^2+^-CaSR^[12,21,42,47,54–56,58,69,249,250]^	VSMC	G_q_/G_i_	Ca^2+^	−
Anti-inflammatory	Adenosine-A_2A_-AR; PGI_2_-IP^[110]^	VSMC	G_s_	PKA	+
	PGI_2_-IP; adenosine-A_2A_AR^[232,239]^	Macrophage	G_s_	PKA	+
	PGI_2_-IP; adenosine-A_2A_AR^[110]^	Fibroblast	G_s_	PKA	+
	PGI_2_-IP; Adenosine-A_2A_-AR^[110]^	EC	G_s_	PKA	+
Pro-inflammatory	ET1-ET_A_; MCP1-CCR2; RANTES-CCR5; TXA_2_-TP^[69,163]^	VSMC	G_q_/G_i_	Ca^2+^	−
	LTB_4_-LTB_4_R; MCP1-CCR2^[163,164]^	Macrophage	G_q_/G_i_	Ca^2+^	−
	PAF-PAFR; TXA_2_-TP^[46,167,169]^	EC	G_q_/G_i_	Ca^2+^	−
Cardiac myocyte hypertrophy	AngII-AT_1_; succinate-GPR91; thrombin-PAR^[205,206]^	Cardiac myocyte	G_q_/G_i_	Ca^2+^	−
Cardiac fibrosis	Thrombin-PAR_1–4_^[223,225]^	Cardiac fibroblast	G_q_/G_i_/G_12_/_13_	Ca^2+^/RhoA	−

+: PH-protective; −: PH-pathogenic; VSMC: vascular smooth muscle cells; EC: endothelial cell

**Table 2.** Current G protein-coupled receptor clinical trials in pulmonary hypertension

Clinical trials name	Sponsor	Drug	Target
Tomorrow	Acetilon	Macitentan	ET_A_/ET_B_ antagonist
ADAPT	United therapeutics		IP agonist
		Orenitram	IP agonist
	Lung biotechnology	BPS-314d oral treprostanil	IP agonist
	Arena pharmaceuticals	APD-811	IP agonist
INSPIRE	Liquidia technologies	Inhaled treprostanil	IP agonist
